# Chitosan Composites for Bone Tissue Engineering—An Overview

**DOI:** 10.3390/md8082252

**Published:** 2010-08-02

**Authors:** Jayachandran Venkatesan, Se-Kwon Kim

**Affiliations:** 1 Department of Chemistry, Pukyong National University, Busan 608-737, Korea; E-Mail: venkatjchem@gmail.com; 2 Marine Bioprocess Research Center, Pukyong National University, Busan 608-737, Korea

**Keywords:** chitosan, hydroxyapatite, carbon nanotube, bone tissue engineering

## Abstract

Bone contains considerable amounts of minerals and proteins. Hydroxyapatite [Ca_10_(PO_4_)_6_(OH)_2_] is one of the most stable forms of calcium phosphate and it occurs in bones as major component (60 to 65%), along with other materials including collagen, chondroitin sulfate, keratin sulfate and lipids. In recent years, significant progress has been made in organ transplantation, surgical reconstruction and the use of artificial protheses to treat the loss or failure of an organ or bone tissue. Chitosan has played a major role in bone tissue engineering over the last two decades, being a natural polymer obtained from chitin, which forms a major component of crustacean exoskeleton. In recent years, considerable attention has been given to chitosan composite materials and their applications in the field of bone tissue engineering due to its minimal foreign body reactions, an intrinsic antibacterial nature, biocompatibility, biodegradability, and the ability to be molded into various geometries and forms such as porous structures, suitable for cell ingrowth and osteoconduction. The composite of chitosan including hydroxyapatite is very popular because of the biodegradability and biocompatibility in nature. Recently, grafted chitosan natural polymer with carbon nanotubes has been incorporated to increase the mechanical strength of these composites. Chitosan composites are thus emerging as potential materials for artificial bone and bone regeneration in tissue engineering. Herein, the preparation, mechanical properties, chemical interactions and *in vitro* activity of chitosan composites for bone tissue engineering will be discussed.

## 1. Introduction

Research on biomaterials for bone implantation and replacement has expanded considerably over the last four decades. In recent years, significant progress has been made in organ transplantation, surgical reconstruction and the use of artificial protheses to treat the loss or failure of an organ or bone tissue. The establishment of a load bearing biomaterial must be incorporated with natural bone. The implanted biomaterial should possess the following criteria: biocompatibility, osteoconductivity, high porosity and biomechanical compatibility [1]. For this requirement, autografts and allografts are used extensively for bone grafts. In the autograft technique, bone from another part will be harvested within the body, and this material fills the gap and provides optimal osteoinductivity, osteoconductivity and osteogenic properties. However, it has its own disadvantages: autografting often leads to complications in wound healing, additional surgery, donor pain and an inadequate supply of bone to fill the gap [2]. In the allograft technique, cadaver bones have been used, but it has problems with immunogenic reactions and the risk of acquiring transmissible diseases (AIDS and hepatitis) from tissues and fluids. These limitations and concerns have created substantial interest in the development of artificial materials as bone graft substitutes [3]. Very few compounds are classified as bioactive, biodegradable and osteoconductive. Chitosan (CTS) and hydroxyapatite (HAp) are among the best bioactive biomaterials in bone tissue engineering and renowned for their excellent biocompatibility with the human body environment [4].

Natural polymer composite materials are becoming increasingly important as scaffolds for bone tissue engineering. Next generation biomaterials should combine bioactive and bioresorbable materials, which mimic the natural function of bone and activate *in vivo* mechanisms of tissue regeneration. Composite materials based on combinations of biodegradable polymers and bioactive ceramics, including CTS and HAp, are discussed as suitable materials for scaffold fabrication. These composites exhibit tailored physical, biological and mechanical properties as well as predictable degradation behavior. The appropriate selection of a particular composite for a given application requires a detailed understanding of relevant cells and/or tissue response. An overview of these findings is presented and discussed in this review, highlighting the influence of material preparation methods, scaffold mechanical strength, *in vitro* activity of scaffold materials and chemical interaction with CTS polymer matrixes. The review also emphasises future artificial bone materials, suggesting the utility of polymer composites in this field of biomaterials science.

Various marine sources polysaccharides have been used for treatment of bone diseases like osteoporosis [5], arthritis [6], and so on. In order to create a moist environment for rapid wound healing, a hydrogel sheet composed of a blended powder of alginate, chitin/chitosan and fucoidan has been developed as a functional wound dressing [7].

## 2. Chitosan for Bone Tissue Engineering

### 2.1. Preparation of chitosan by chemical methods

**Figure 1:**
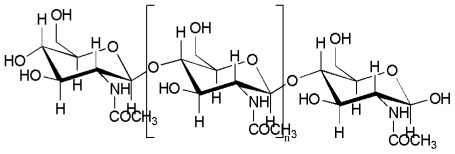
Structure of chitin.

Chitin (Figure 1) is the second most abundant natural polymer after cellulose. CTS is produced from chitin, which is a natural polysaccharide found in crab, shrimp, lobster, coral, jellyfish, butterfly, ladybug, mushroom and fungi. However, marine crustacean shells are widely used as primary sources for the production of CTS [8,9]. Crab and shrimp are important marine species of great commercial importance in the tropical and subtropical waters of the Pacific, Atlantic and Indian oceans. The waste from crab and shrimp processing has recently become a serious issue in coastal areas. Selective isolation of bioactive material from these wastes is the simplest way to decrease the pollution. It not only reduces the environmental pollution because of the disposal of this under utilized by products of crabs and shrimps, but also increases the potential applications of CTS. Moreover, the chemical hydrolysis and enzymatic methods, widely used for the isolation for CTS from marine crustaceans shell, are quite inexpensive.

**Figure 2:**
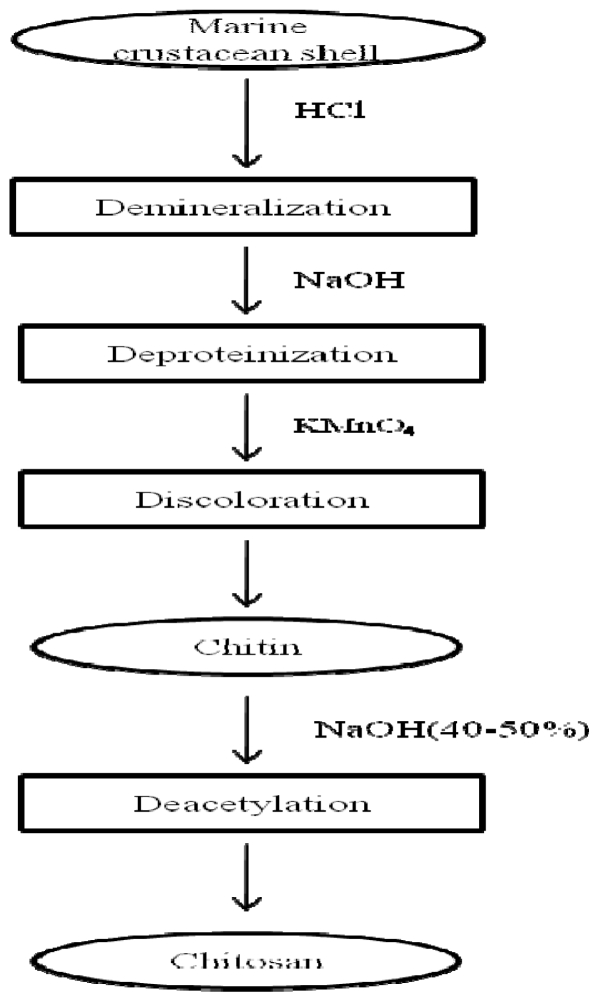
Preparation of chitin and chitosan from marine crustaceans.

In chemical hydrolysis method, four main steps are involved in order to produce CTS from marine crustacean shell as depicted in Figure 2. They are (i) demineralization; (ii) deproteinization; (iii) discoloration and (iv) deacetylation. To produce 1 kg of 70% deacetylated CTS from shrimp shells, 6.3 kg of HCl and 1.8 kg of NaOH are required [8,9].

### 2.2. Enzymatic hydrolysis method

Chitosan can be isolated directly from the cell walls of certain fungi, but commercially available CTS are usually prepared from chitin. CTS can be produced via traditional chemical method, however, problems exist related to poor quality and environmental chemical pollution [10]. The enzymatic method could provide an alternative to the current chemical production method. The degree of deacetylation and molecular weight of the CTS depends on the source and preparation method (molecular weight ranges from 300 to over 1,000 kDa, degree of deacetylation from 30% to 95%). CTS can be degraded by enzyme and form various small molecular weight fragments. Chitosanase has been used for the preparation of small molecular weight CTS with various methods such as batch reactor, column reactor and ultrafiltration membrane reactor [11]. The CTS has been extracted with various enzymes like lysozyme, snailase, neutral protease and novel chitin deacetylase from *Scopulariopsis brevicaulis*. The average molecular weight of CTS obtained using the enzymatic method (267.97 kDa) has been reported to be far greater than that obtained by chemical method (84.04 kDa) [10,12].

### 2.3. Properties and application of chitosan

**Figure 3:**
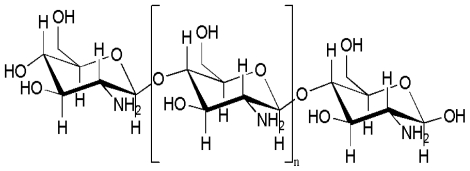
Structure of fully deacetylated chitosan.

CTS is a copolymer consisting of β-(1→4)-2-acetamido-d-glucose and β-(1→4)-2-amino-d-glucose unit linkages (Figure 3) [13,14]. Over the past two decades, CTS has been developed considerably in biomedical applications due to its high biocompatibility, biodegradability, porous structure, suitability for cell ingrowth, osteoconduction and intrinsic antibacterial nature [15], CTS offers a wide range of applications, including cartilage tissue engineering [16], wound healing [17] and orthopedic applications [15]. Degradable polymeric implants eliminate the need for a second surgical operation and can prevent some of the problems associated with stress shielding during post-healing, and can also be used simultaneously to deliver therapeutic drugs to treat infections or growth factors to accelerate new bone growth [18]. There is a growing interest in exploiting the field of bone tissue engineering for composite preparation. This has created a wide range of application in the preparation of artificial organs.

CTS can be easily modified into various forms like films, fibers, beads, sponges, and more complex shapes for orthopedic treatment [15]. The cationic nature of CTS is responsible for attracting various negative charged proteoglycans. Porous materials have a highly significant role in the bone implantation process. Porous CTS structures can be formed by freezing and lyophilizing CTS acetic acid solutions in suitable molds [19,20]. CTS have been combined with a variety of materials such as HAp, alginate, hyaluronic acid, calcium phosphate, poly (methyl methacrylate), poly-l-lactic acid and growth factors for potential application in orthopedics.

## 3. Composite Materials for Bone Tissue Engineering

Composite materials are now playing predominant role as scaffolds in bone tissue engineering. CTS has numerous advantageous properties for orthopedic applications, as described above and elsewhere [15], which make it ideal as a bone graft substituent. CTS scaffolds are flexible and their mechanical properties are inferior to those of normal bone, as it is unable to support load bearing bone implants. Moreover, CTS itself is not osteoconductive, although addition of ceramic materials improves its osteoconductivity and mechanical strength.

CTS scaffolds alone cannot imitate all the properties of natural bone. The substantial development of composite materials with CTS mimics all the properties of bone. As proven, calcium phosphate materials are osteoconductive to mimic the inorganic portion of natural bone, while CTS/HAp composite materials show promise in mimicking the organic portion as well as the inorganic portion of natural bone. Several studies have been conducted with CTS/HAp composite materials for bone tissue engineering [15,18,21–30]. Calcium phosphate compounds are of great interest in the field of bone tissue engineering. Hydroxyapatite [Ca_10_(PO_4_)_6_(OH)_2_] is one of the most stable forms of calcium phosphate and it occurs in the bone as a major component (60 to 65%) [31]. HAp also possesses a variety of uses, including orthopedic, dental and maxillofacial applications. Therefore, HAp has recently emerged as an important compound for artificial bone preparation. It stimulates osteoconduction being gradually replaced by the host bone after implantation. It is being used for orthopedic replacements, especially in bone regeneration and dental implant treatment. The mechanical properties of HAp are poor, though, so it cannot be used for load bearing bone tissues. Polymers have been used to improve the mechanical properties of HAp (compressive strength, Young’s modulus, fracture toughness) [32]. When CTS is combined with HAp, it might be able to mimic the function of natural bone.

### 3.1. Preparation of CTS/HAp composite materials

Numerous methods have been used to prepare CTS/HAp composites, especially for nanocomposite preparation, which play an excellent role in the extra-space arrangement in the matrix and have the best biomedical properties and biomaterial applications [22]. CTS/HAp nanocomposite with a homogeneous microstructure has been prepared using *in situ* synthesis [33], precipitation method [34], *in situ* co-precipitation synthesis with an electrospinning process [21,23,30,35,36], simple *in situ* hybridization [18], solvent casting and evaporation method [24], *in situ* chemical method [25,37], freezing and lyophilization [26,27], combined sintering and freeze-drying technique [28], self-assembly of static electricity [38], simple mixing and heating method [29], biomimetic method [39–41], low temperature wet chemical method [42], thermally induced phase separation technique [43], dual membrane diffusion system [44], electrochemical deposition [45], electrochemistry assisted deposition [46,47], electrophoretic deposition [48,49], natural HAp mixed with CTS [50], double diffusion technique [51] and wet spinning method [52].

### 3.2. Mechanical properties of CTS/HAp composite

The mechanical properties of the CTS/HAp composites play a significant role in bone tissue engineering. The intermolecular hydrogen bond and chelate interaction between the CTS and HAp contribute to good mechanical properties. There is a possible interaction between the NH_2_ group and primary and secondary –OH group of CTS with Ca^2+^ (metal coordination interaction) of HAp. The possible interaction between CTS and HAp is discussed in a later section. This interaction might be responsible for the higher mechanical strength of the composite scaffolds as compared to CTS and HAp alone. Compressive strength has been a widely used parameter to find out the mechanical strength of porous scaffolds. Li *et al.* compared the compressive strengths of different composite ratio of CTS/HAp and found the maximum compressive strength to be 119.86 MPa (30:70 CTS/HAp ratio). Increasing the HAp ratio leads to an increase in the compressive strength [53]. The incorporation of CTS into HAp matrix via blending methods would result in the decrease of mechanical properties of composite material due to the weaker interfacial bonding between CTS and HAp matrix and as a result, the compressive strength reduces to 47.8 MPa [54]. The molecular weights of CTS also contributes significantly to the mechanical properties. In general, the high molecular weight CTS scaffolds have higher compression modulus than medium molecular weight CTS. The compression moduli of high molecular CTS and CTS/nHAp (1%) nanocomposite scaffold were found to be 6.0 ± 0.3 kPa and 9.2 ± 0.2 kPa, respectively [27]. The mechanical properties of CTS composite scaffold also depend on the temperature and it increases with an increase in the temperature. A possible rationale to this may be that with an increase in the temperature, the interfacial bonding between CTS and HAp increases [30]. Water content of the scaffold may also have a major role to play in the mechanical strength. In a study, it was found that the nHAp/CTS/carboxymethyl cellulose (40/30/30% w/v) has highest mechanical property of 40 MPa in dry and 12 MPa in wet state [38].

### 3.3. *In vitro* study of CTS/HAp composites

CTS have been widely used in orthopedic treatment since it was shown to promote osteoblastic cell growth. When CTS is incorporated with HAp, it remarkably increases the osteoblastic cell growth on the scaffolds [15,21,24,25,27]. The 3-(4,5-dimethylthiazol-2-yl)-2,5-diphenyltetrazolium bromide (MTT) method has been frequently used to determine the cytotoxicity of CTS composite materials. All the scaffolds’ results showed that the CTS/HAp composite had no cytotoxicity with positive cell attachment and proliferation growth of the osteoblast cells [15,23–25,50,51]. The composite scaffolds possess a pore size of 100–200 μm, providing a spatial arrangement of cells (10–30 μm) and thus cells are able to migrate towards the composite [55]. It was observed that when the osteoblast cells were cultured in the medium of phosphorylated CTS/HAp, the cell morphology changed within 30 min of seeding and later became triangular at 24 h, polygonal at 48 h, and finally, aggregated to be indistinct at 5 days [56]. Apart from HAp, other calcium phosphate minerals also provide adhesion and cell proliferation when combined with CTS. The cell proliferation and adhesion has been found with osteoblast mouse cells MC3T3-E1 and L929 cells in CTS/calcium phosphate specimens [28,57].

The alkaline phosphatase activity (ALP) is considered to be an important marker of the differentiation of osteoblast cells at a relatively early bone forming stage and has been widely used to evaluate ALP for scaffold materials. The CTS/HAp composite scaffolds possess higher ALP activity compared to the CTS scaffold whereas the highest ALP activity has been achieved in the composite containing 30–40% of HAp with good cell proliferation; however, cell proliferation decreases with an increase in the HAp concentration [21,51,58]. The modified CTS and its composites are found to have good cell proliferation and higher ALP activity then compared to non modified CTS. CTS glutamate and HAp containing cultured osteoblasts were found to be promising biomaterials for repair bone defects *in vivo* [56,59].

### 3.4. Chemical interaction of CTS/HAp composites

**Figure 4:**
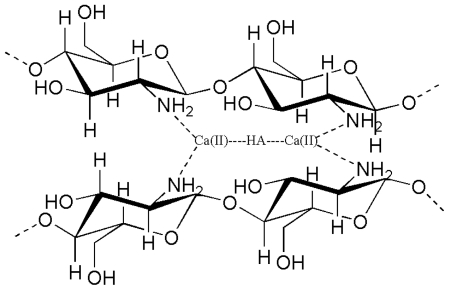
Chemical interaction between CTS-Hap.

Ca^2+^ ions appear on the terminated surface of HAp crystals, which have coordination number of seven and are strictly held in the structure (Figure 4). Therefore, there is a possibility to form coordination bonds between the -NH_2_ of CTS and Ca^2+^ of HAp [24,60].

## 4. Carbon Nano Tubes for Bone Tissue Engineering

Carbon nanotubes (CNTs) are allotropes of carbon with a cylindrical nanostructure and constructed with length-to-diameter ratio of up to 28,000,000:1. These cylindrical carbon molecules have novel properties, which make them potentially useful in many applications in nanotechnology, electronics, optics and materials sciences. CNTs have a high Young’s modulus (1.0–1.8 TPa), high tensile strength (30–200 GPa) and high elongation at break (10–30%). In addition, they have extremely small size (about 1–10 nm in diameter), high aspect ratio (>1,000), high structural and chemical stability, and stiffness, as well as remarkable electrical, thermal, optical and bioactive properties [61,62]. All these properties make CNTs especially promising candidates as reinforcement fillers in the development of nano composites. It has been observed that the combination of CNT with CTS leads to an enormous increase in the mechanical strength of the composite [63].

CNTs hold great interest with respect to biomaterials, particularly those to be positioned in contact with bone such as prostheses for arthroplasty, plates or screws for fracture fixation, drug delivery systems, and scaffolding for bone regeneration. The most important concerns for the use of CNTs as a biomaterial are tissue safety, but only few reports have addressed the toxicity of CNTs. In particular, bone tissue compatibility is extremely important for using CNTs in biomaterials. Usui *et al.*, who first developed CNT, found that these tubes have good bone tissue compatibility and are capable of permitting bone repair and becoming closely integrated with bone tissue and accelerate bone formation stimulated by recombinant human bone morphogenetic protein-2 [64].

A big challenge in the bone tissue engineering is mechanical strength improvement of the scaffold materials. One of the main purposes of creating CTS composites is to improve the mechanical strength of the material. CNT is a promising material to fulfill that gap due to its strong mechanical properties. Several authors have developed many composites as well as scaffold materials with CNT. It has been observed that cell adhesion on multi-walled CNT (MWCNT) coated dish is much higher than that on the collagen coated dish [65].

### 4.1. Preparation of CTS/CNT composite

One of the major problems in the preparation of CTS with CNT materials is the difficulty to disperse the CNTs well in the polymer matrix. Several methods have been used for this purpose such as controlled surface deposition and cross linking process [66], simple solution evaporation method [63], thermally induced phase separation followed by freeze drying [67], electrodeposition [68], spray layer by layer technique [69], pH and electrical actuation [70] and wet spinning method [71].

### 4.2. Mechanical properties of CTS/CNT composite

Wang *et al.* reported that CNT is homogeneously dispersed throughout the CTS matrix. When 0.8% of CNT was introduced in the CTS matrix, the mechanical properties, including the tensile modulus and strength of the nanocomposite were greatly improved by about 93% and 99% [63]. A small addition of CNTs significantly improves the tensile properties of CTS matrix, and the mechanical properties increase with the increase of CNTs’ loading (0.4 wt% of CNTs filler) whereas the tensile modulus and strength of the nanocomposite increases dramatically by about 78% and 94%. Due to the aggregation of CNTs within the CTS matrix at higher concentrations, with further increases of the loading level of CNTs, the tensile modulus only increases slightly, while the tensile strength remains stable. For instance, with 2.0 wt% of MWNTs filler, the tensile modulus of the CTS matrix is 2.15 GPa, only 0.07 GPa higher than that of 0.8 wt% of MWNTs. The tensile test results indicate the mechanical properties of the CTS/CNTs [63].

In another study, the tenacity of neat CTS fiber was recorded as 96 MPa, whereas that of CTS reinforced with purified but non-functionalized single-walled CNTs (p-SWCNTs) increased from 132 MPa for 0.01 wt% SWCNT addition to 180 MPa for 0.4 wt% SWCNT addition. Even at very low SWCNT concentration (0.01 wt%), a significant increase in tenacity was observed. On the other hand, on incorporation of functionalized CNT (f-CNT), the tenacity increased from 136 MPa for 0.01 wt% SWCNT addition to 226 MPa for 0.4 wt% addition. This continuous improvement in tenacity (even at higher SWCNT concentrations) may be ascribed to better dispersion as well as interaction of functionalized nanotubes in CTS [70]. This might be because of the strong chemical interaction as functionalized CNT’s COOH group interacts with amine group of CTS matrix.

### 4.3. *In vitro* study of CTS/CNT composites

CNTs are like an inert matrix material in which cells can proliferate in easily. Zanello *et al.* suggested that CNTs can be used as an alternative material for the treatment of bone pathologies with the potential for the regrowth of normal bone. Osteoblast-like cells were grown on electrically neutral CNTs and they were also found to produce mineralized bone [72]. The impurities in commercial SWCNTs, such as metal catalysts and amorphous carbon particles, are reported to be toxic to cells and could induce intracellular reactive oxygen species. However, the addition of CTS with amine functional groups should decrease the cytotoxicity. Compared to p-SWCNTs, f-SWCNT grafted with CTS provides better cell proliferation and cell attachment. An explanation to this may be that the cellular membranes are negatively charged, and thus cells can more easily attach and grow on more positively charged surfaces [73].

Wörle-Knirsch *et al*. suggested that the MTT method is not reliable for finding out the cytotoxity of CNT based materials, as the MTT formazan crystals formed in the MTT reaction are lumped. As a consequence, false results may show a strong cytotoxic effect in the MTT assay after 24 h and show that roughly 50% of the cells die. But other methods, such as WST-1, lactate dehydrogenase, fluorescence-activated cell sorting assisted mitochondrial membrane potential determination and annexin-V/PI staining revealed no cytotoxicity [74]. Very little work has been done with CTS/CNT composite materials for bone tissue engineering. CNT can mimic the strength of natural bone in composite or scaffold materials. Research strategies still need to be developed in the field of CTS/CNT composite material preparation and *in vitro* as well as *in vivo* activity of CNT based polymeric materials.

### 4.4. Chemical interactions between CTS/CNT

**Figure 5:**
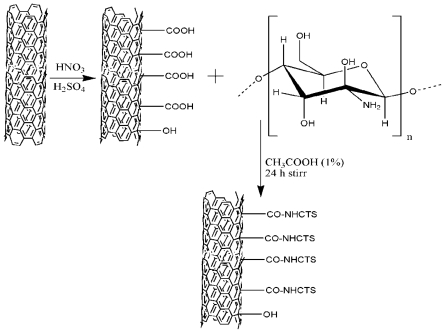
Chemical interactions between CTS and CNT.

The raw CNTs are mainly hydrophobic and poorly miscible with water. The acid treated CNTs contain many defects and hydrophilic groups, such as -OH and -COOH, which are very helpful for improving the solubility of CNTs in water. CTS, a hydrophilic biopolymer, possesses three kinds of functional groups *viz*. amino, primary, and secondary hydroxyl groups in a glucosamine unit, and the functionalized CNTs contain carboxylic and hydroxyl groups. There is great possibility that strong hydrogen bonds may form between chitosan and the CNTs (Figure 5). The compatibility and strong interaction between MWCNTs fillers and the matrix greatly enhances the dispersion as well as the interfacial adhesion, thus significantly increasing the mechanical properties of the matrix [63]. The covalent functionalisation of CTS and CNT improves the interaction between CTS with CNT, reduces the damage of CNT and increases its mechanical properties.

## 5. Remaining Challenges and Future Directions for Bone Biomaterials

The organic portion plays a major role in bone implants, therefore their matrices are often hybrid in composition. Apart from CTS, HAp and CNT, large numbers of synthetic polymers have been used for the preparation of composite materials for artificial bone. It was observed that they showed better results in the case of cell viability, cell proliferation, alkaline phosphate activity and mineralization assays [75]. Several synthetic polymers have been used to prepare artificial scaffolding materials but have a number of disadvantages. One of the major drawbacks of synthetic polymers are their degradation times which are half than that of a natural polymer. Nowadays, synthetic polymers like poly lactic acid, poly-l-lactide acid, polyethylene glycol, polyhydroxyl ethyl methacrylate with carbon nano tubes [76], poly caprolactone and polylactic-co-glycolic acid are widely used for scaffold materials. The synthetic polymer not only increases the scaffold strength, but also acts as a drug delivery device to induce bioactivity. Whether synthetic polymers would be able to reinforce the mechanical properties of HAp, is still a burning question.

Even though CNT have tremendous mechanical strength and are able to mimic the natural bone function, the uniform dispersion of CNT in the CTS and HAp matrix still remains a challenge. CNTs are not easily dispersed and bundles form in the polymer matrix. CNT should also be in a very pure form for biomedical applications as metal-containing CNTs are toxic to cells, and the production of pure forms of CNTs is another challenge. The cytotoxity of CNT is still a controversial topic due to errors in cytotoxic analysis techniques, as reported earlier, and the toxicity of CNT might vary depending on the cell type, purity of the CNTs and functionalisation.

## 6. Conclusions

Composite CTS-based materials have been found to have a predominant role in bone tissue engineering in recent years. The combination of biocompatible polymers and bioresorbable ceramic materials can mimic the natural function of bone. CTS with HAp composites are found to be potential bone implant materials with good osteoconductive, osteoinductive and osteogenic properties. The structural, mechanical, chemical interaction and *in vitro* study of CTS, HAp and CNT have been carried out for bone tissue engineering. Although many CTS/HAp composite materials have been developed, problems persist with their mechanical properties. Hence, much research is in progress to address the gap in the development of mechanical properties. Much research work is still needed to address the cytotoxicity of CNTs. Though challenges still exist, the addition of CNT to improve the mechanical properties of CTS and ceramic (HAp) composite would surely support and stimulate the function of natural bone. The development of research on the efficacy of CTS composite will open great possibilities for future bone tissue engineering.
